# Evaluation of the Course and Outcome of Aggressive Retinopathy of Prematurity

**DOI:** 10.7759/cureus.84589

**Published:** 2025-05-21

**Authors:** Shreya Raj, Vivek Som, Kavita Kumar

**Affiliations:** 1 Ophthalmology, Gandhi Medical College, Bhopal, IND

**Keywords:** anti-vegf, a-rop, blindness, gestational age, oxygen supplementation

## Abstract

Background: This study was conducted to evaluate the course and outcome of aggressive retinopathy of prematurity (AROP) and to study the risk factors associated with AROP.

Methodology: This was a prospective observational study, conducted in the Department of Ophthalmology, Gandhi Medical College, Bhopal, India, from August 2022 to June 2024 on preterm infants. Infants were examined in the retinopathy of prematurity (ROP) screening cubicle using aseptic precautions. All ocular findings were recorded on the proforma, and any abnormal findings were confirmed by the study guide. In cases where ROP is detected, fundus photo documentation is done. Counselling of the parents was conducted, emphasizing the importance of timely follow-up and the need for periodic reviews.

Results: The incidence of ROP was 24.9%, while the incidence of AROP was 10.5%. Overall, AROP was detected in 107 eyes of 56 patients. Zone I was most commonly involved in 58 eyes (54.2%), followed by posterior zone II in 49 eyes (45.8%). We documented a significant association of AROP with prolonged duration of stay in the neonatal intensive care unit (NICU) (18.20±10.21 vs. 13.17±6.35 days), oxygen supplementation of more than one week (87.5% vs. 68.4%), prolonged duration of oxygen supplementation (16.50±8.34 vs. 10.50±5.2), and ventilatory support of more than one week (16.1% vs. 3.9%; p<0.05). We found regression following treatment with anti-vascular endothelial growth factor (anti-VEGF) in significantly higher proportions of cases with zone I AROP and progression following anti-VEGF in cases with zone II posterior AROP (p<0.05).

Conclusions: The relatively high incidence of AROP, predominantly bilateral and affecting zone I, underscores the need for vigilant screening and early intervention in high-risk infants. Our findings reinforce the importance of minimizing exposure to supplemental oxygen and optimizing neonatal care practices to reduce the risk of AROP development and progression.

## Introduction

Retinopathy of prematurity (ROP) is a pathological condition that poses a significant risk to vision and is widely recognized as a leading cause of childhood blindness on a global scale since the 1950s [1]. According to available estimates, the occurrence of blindness in children worldwide due to this particular condition has been documented to be a minimum of 50,000 cases [2]. ROP is a medical disorder characterized by the abnormal growth of blood vessels in the underdeveloped retina of premature infants. It has a varied progression and outcome. Depending upon the severity of the disease, it is classified into five stages and, depending on location, into three zones. Despite the positive results shown in the management of preterm newborns, ROP continues to be a significant contributor to visual impairment and blindness among children [3].

The ideas of ROP zone, stage, and plus illness have been significantly impacted by advancements in retinal imaging techniques [4-6]. The term "posterior zone II" is defined by the International Classification of Retinopathy of Prematurity, Third Edition (ICROP3), as an area located two disc diameters between zone I and zone II. ICROP3 also characterizes a range of vascular changes from normal to plus disease and suggests the adoption of the term "aggressive ROP" instead of "aggressive posterior ROP" [7]. Aggressive retinopathy of prematurity (AROP), previously referred to as aggressive posterior retinopathy of prematurity (APROP), typically manifests in infants born prematurely, namely, those with a gestational age of fewer than 28 weeks and a birth weight of ≤1000 g [8]. However, AROP is increasingly recognized to also occur in larger preterm infants and beyond the posterior retina, particularly in regions of the world with limited resources [7]. AROP refers to an expeditiously advancing manifestation of ROP. Signs of AROP include the rapid development of stage 3 with plus disease, extremely anomalous vasculature with shunting and vessel loops present, and flat-appearing stage 3 without line or ridge demarcation [1].

The clinical manifestations exhibit distinct characteristics that distinguish them from the traditional presentation of ROP. In the event that timely intervention is not administered, the illness has the potential to advance swiftly to stage 5 ROP. Timely screening helps us to diagnose and give timely and appropriate treatment to preserve vision and prevent blindness. The most effective management strategy is a subject of debate, considering the advantages and disadvantages associated with both laser photocoagulation and intravitreal anti-vascular endothelial growth factor (anti-VEGF) injections [9]. In this context, this prospective observational study was conducted to study and evaluate the course and outcome of AROP and to study the risk factors associated with AROP.

## Materials and methods

This study was conducted as a facility-based prospective observational study in the Department of Ophthalmology, Gandhi Medical College, Bhopal, India, from August 2022 to June 2024. All the infants with a birth weight of 2000 g or less and/or a gestational age of 37 weeks or less, irrespective of gender, referred from Pediatric Medicine or peripheral region to ROP clinic and cases or parents giving consent were included, whereas all term neonates, with any ocular congenital anomaly and ocular abnormality other than ROP, were excluded from the study.

Before commencing the study, ethics committee approval was obtained from the Institutional Ethics Committee of Gandhi Medical College (approval number: 83/IEC/2022). Written informed consent was taken from the parents/guardians of the cases, and confidentiality of the cases was maintained.

All eligible neonates attending the ROP clinic for screening who fulfilled the inclusion and exclusion criteria were enrolled. Parents or guardians were briefed about the examination procedure in their native language, and written informed consent was obtained before the procedure. Comprehensive demographic details were obtained in a proforma. Antenatal history, involving details about hypertensive disorders of pregnancy, diabetes mellitus, premature rupture of membranes, cervical incompetence, previous instances of premature birth, and mode of delivery, was gathered. Apart from this, postnatal history, including apnea/asphyxia, respiratory distress syndrome and management, neonatal intensive care unit (NICU) hospitalization and duration, oxygen saturation, supplementation, and duration, sepsis, hyperbilirubinemia, thrombocytopenia, and blood transfusion, was also documented.

Infants were examined in the ROP screening cubicle, with precautions taken to prevent hypothermia, including avoiding air conditioning and fans. Babies were securely wrapped with a sterile cloth to facilitate examination by restricting movements and to maintain warmth. Aseptic precautions were diligently observed, involving hand washing and sterilization of pediatric wire speculums and scleral indenters before fundus examination. Proper anterior segment examination was conducted, and findings were documented. Pupils were dilated using topical 2.5% phenylephrine and 0.5% tropicamide at an interval of 10-15 minutes. Posterior segment examination was performed using a pediatric lid speculum, a binocular indirect HEINE ophthalmoscope (HEINE Optotechnik, Gilching, Germany), and a 28-diopter lens. The retinal periphery was examined using a scleral indenter.

**Figure 1:**
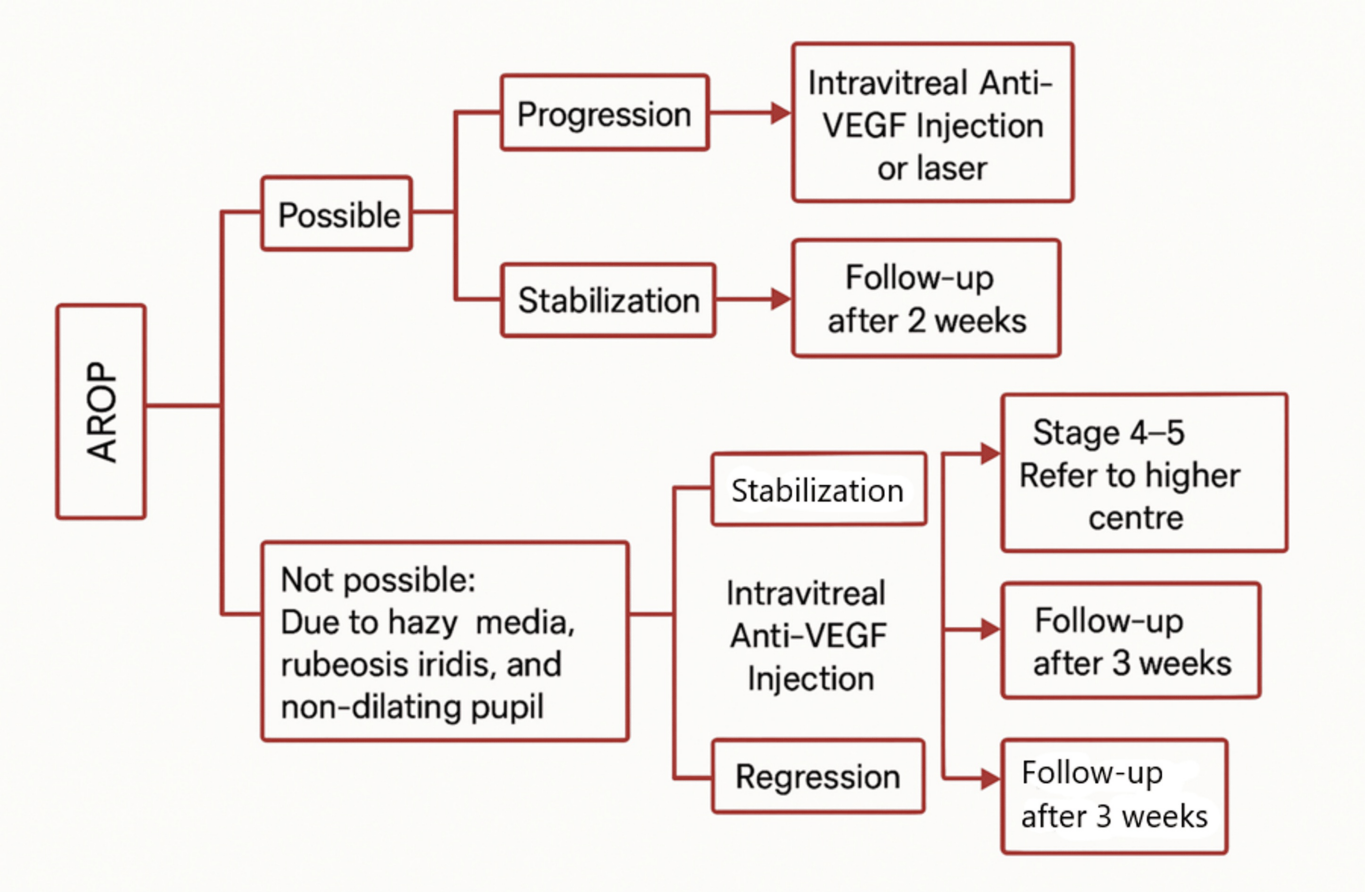
Flowchart of the present study AROP: aggressive retinopathy of prematurity; anti-VEGF: anti-vascular endothelial growth factor

After the complete examination, the speculum was gently removed, and moxifloxacin 0.5% antibiotic eye drops were instilled. All ocular findings were recorded on the proforma, and any abnormal findings were confirmed by the study guide. The zone of vascularization (from I to III) and the stage of ROP (stages 1-5) were documented as per the ICROP3 [7]. Any patient who comes under zone II and stage mentioned as per ICROP3 at presentation is included in the study. Now, the cases which are selected and included in the study were divided as follows: group 1, AROP, and group 2, treatment requiring non-AROP (Figure 1).

In cases where ROP is detected, fundus photo documentation is done using 3nethra NeoCam (Forus Health Pvt. Ltd., Bengaluru, India) for future reference and to detect the regression or progression of ROP either spontaneously or following treatment. Counselling of the parents was conducted, emphasizing the importance of timely follow-up and the need for periodic reviews.

All the hematological and other required investigations, such as complete blood profile and coagulation profile, needed for the management of the patient by the pediatrician were done. The neonates were treated as per the standard treatment guidelines, given by the Early Treatment for Retinopathy of Prematurity (ETROP). The treatment procedure given was noted as follows: laser, anti-VEGF, anti-VEGF+laser, and surgery. These patients were followed up.

Statistical analysis plan

All the variables were grouped as per mathematical transformation into nominal/ordinal/interval and ratio. Point estimates with dispersion measures were calculated using Microsoft Excel (Microsoft Corporation, Redmond, Washington, United States) and IBM SPSS Statistics for Windows, Version 27.0 (Released 2020; IBM Corp., Armonk, New York, United States). The extent of type 1 error was measured through parametric analysis. The t-test was used to identify any significant difference among the detected proportions and means. The chi-squared test was applied at appropriate places. Statistical tools, such as IBM SPSS Statistics for Windows, Version 20.0 (Released 2011; IBM Corp., Armonk, New York, United States), and MedCalc for Windows, Version 19.5 (MedCalc Software, Ostend, Belgium), were employed for the analysis.

## Results

In the present study, a total of 530 neonates were screened, and of them, 132 neonates with ROP were enrolled in our study. The incidence of ROP was 24.9%, while the incidence of AROP was 10.5%. Overall, AROP was detected in 107 eyes of 56 patients. Among them, 51 cases (91.1%) had bilateral AROP, and five (8.9%) had unilateral AROP. Zone I was most commonly involved in 58 eyes (54.2%), followed by posterior zone II in 49 eyes (45.8%). Table 1 provides the characteristics of the study population.

**Table 1:** Characteristics of the study population The p-value was calculated using the chi-squared test and one-way ANOVA for proportions and means, respectively. A p-value of <0.05 is considered significant. AROP: aggressive retinopathy of prematurity; NA: not applicable

Baseline variables	AROP (n=56)	Non-AROP (n=76)	χ^2^	F-value	P-value
Gender (M:F)	30:26 (53.6%:46.4%)	38:38 (50%:50%)	0.165	NA	0.685
Gestational age (weeks)	31.23±2.85	31.75±2.80	NA	1.04	0.299
Birth weight (kg)	1.21±0.27	1.29±0.33	NA	1.44	0.152
Residence (urban:rural)	10:46 (17.9%:82.1%)	8:68 (10.5%:89.5%)	1.47	NA	0.225

We documented a significant association of AROP with prolonged duration of stay in the NICU (18.20±10.21 days in the AROP group and 13.17±6.35 days in the non-AROP group), oxygen supplementation of more than one week (87.5% vs. 68.4%), prolonged duration of oxygen supplementation (16.50±8.34 vs. 10.50±5.2), and ventilatory support of more than one week (16.1% vs. 3.9%; p<0.05). However, we found no significant association of AROP with other maternal and neonatal risk factors depicted in Table 2.

**Table 2:** Association of cases with AROP with maternal as well as neonatal risk factors The p-value was calculated using the chi-squared test and one-way ANOVA for proportions and means, respectively. A p-value of <0.05 is considered significant. AROP: aggressive retinopathy of prematurity; LSCS: lower segment cesarean section; H/O: history of; PROM: premature rupture of membranes; GA: gestational age; BW: birth weight; RDS: respiratory distress syndrome; NICU: neonatal intensive care unit; wk: week; NIV: non-invasive ventilation; NA: not applicable

Risk factors		AROP (n=56)	Non-AROP (n=76)	χ^2^	F-value	P-value
Maternal factors	Mode of delivery (LSCS vs. vaginal)	16/40 (28.6% vs. 71.4%)	18/58 (23.7% vs. 76.3%)	0.403	NA	0.53
	Hypertension	6 (10.7%)	8 (10.5%)	0.001	NA	0.972
	Diabetes	3 (5.4%)	8 (10.5%)	1.13	NA	0.29
	Cervical incompetence	3 (5.4%)	5 (6.6%)	0.085	NA	0.77
	H/O premature birth	1 (1.8%)	5 (6.6%)	1.71	NA	0.19
	PROM	1 (1.8%)	0 (0%)	1.37	NA	0.24
	Multiple pregnancy	14 (25%)	10 (13.2%)	3.04	NA	0.08
Neonatal factors	GA (weeks)	31.23±2.85	31.75±2.80	NA	1.04	0.299
	BW (kg)	1.21±0.27	1.29±0.33	NA	1.44	0.152
	Apnea	8 (14.3%)	19 (25%)	2.27	NA	0.13
	Asphyxia	8 (14.3%)	14 (18.4%)	0.397	NA	0.53
	RDS	30 (53.6%)	39 (51.3%)	0.066	NA	0.79
	NICU hospitalization (days)	18.20±10.21	13.17±6.35	NA	3.47	0.001
	Oxygen supplementation <1 wk vs. >1 wk	7/49 (12.5% vs. 87.5%)	24/52 (31.6% vs. 68.4%)	6.53	NA	0.011
	Duration of oxygen supplementation (days)	16.50±8.34	10.50±5.2	NA	5.09	0.001
	Ventilatory support <1 wk vs. >1 wk	47/9 (83.9% vs. 16.1%)	73/3 (96.1% vs. 3.9%)	5.74	NA	0.017
	Ventilator: NIV	20 (35.7%)	16 (21.1%)	3.49	NA	0.062
	Duration of ventilatory support	2 (0-5.75)	0 (0-2)	0.024	NA	0.98
	Hyperbilirubinemia	7 (12.5%)	14 (18.4%)	0.85	NA	0.36
	Thrombocytopenia	2 (3.6%)	7 (9.2%)	1.61	NA	0.20
	Septicemia	8 (14.3%)	13 (17.1%)	0.19	NA	0.66
	H/O blood transfusion	15 (26.8%)	14 (18.4%)	1.32	NA	0.25
	Number of blood transfusions	0 (0-1)	0 (0-0)	0.429	NA	0.668
	Post-menstrual age at presentation	35.41±3.62	35.32±2.89	NA	0.17	0.86

**Figure 2:**
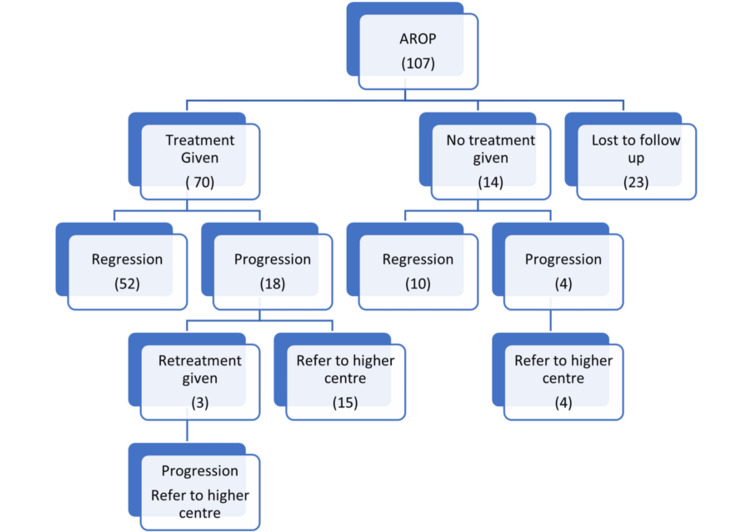
Distribution of eyes according to the type of treatment given and outcome in such cases Median (IQR): A non-parametric test, that is, the Mann-Whitney U test, was applied. AROP: aggressive retinopathy of prematurity

Treatment was given in 70 eyes, and the most common treatment given was laser (n=42), followed by intravitreal anti-VEGF (n=16). However, laser with intravitreal anti-VEGF was given in 12 eyes. Out of 42 cases managed with laser, regression of AROP was noted in 32 eyes, whereas in 10 eyes, progression of AROP was noted. Out of 16 and 12 eyes managed with intravitreal anti-VEGF and combination therapy (laser with intravitreal anti-VEGF), progression was noted in four and four eyes, respectively (Figure 2).

As observed from Table 2, we found regression following treatment with anti-VEGF in significantly higher proportions of cases with zone I AROP and progression following anti-VEGF in cases with zone II posterior AROP. The observed association of zone-wise treatment with outcome was statistically significant (p<0.05). Table 3 provides the details on the association of the treatment provided with the outcome.

**Table 3:** Association of treatment with outcome in patients based on zone The p-value was calculated using the chi-squared test. A p-value of <0.05 is considered significant. anti-VEGF: anti-vascular endothelial growth factor

Zone	Outcome (progression/regression)	Laser	Intravitreal anti-VEGF	Laser+intravitreal anti-VEGF	χ^2^	P-value
I	Progression	6	0	2	138.1	<0.001
	Regression	16	10	4		
II posterior	Progression	4	4	2	126.3	<0.001
	Regression	16	2	4		

## Discussion

AROP is a severe form of ROP characterized by the rapid progression and potential for significant visual impairment in preterm infants. The present study aimed to evaluate the course and outcome of AROP in a fraction of infants at our institution. This investigation sought to identify the incidence of AROP, delineate its clinical presentation, and assess the effectiveness of various treatment modalities in our specific population. The study further aimed to explore the potential risk factors associated with the development and progression of AROP, with a particular focus on both maternal and neonatal factors.

The incidence of ROP (24.9%) and AROP (10.5%) observed in our study aligns with the global trends, highlighting the growing concern of ROP in middle-income countries, as indicated by the "third epidemic" described in the literature [2]. Our AROP incidence is relatively high compared to some studies, such as Gunay et al., who reported a 0.11% APROP incidence in a Turkish tertiary care centre [10]. This discrepancy might be attributed to differences in population characteristics, screening practices, and redefined definitions of AROP and APROP. Notably, our study included a higher proportion of infants with low birth weight and extreme prematurity, known risk factors for APROP when compared with the studies by Kumar et al. (4.7% of severe ROP) and Kim et al. [11,12]. Additionally, the evolving classification of AROP, with the introduction of "aggressive ROP" in ICROP3, may contribute to variations in reported incidence rates [7].

Our study found that zone I was the most commonly involved location in AROP (54.2%), followed by posterior zone II (45.8%). This distribution aligns with the definition of AROP [7], which emphasizes involvement of the posterior retina. The predominance of zone I involvement underscores the severity of AROP in our cohort, as this zone is closest to the macula, the central area of vision. The involvement of posterior zone II is also significant, as it represents a transitional area between the central and peripheral retina, potentially impacting both visual acuity and peripheral vision [9].

In our study, we did not find a significant association between AROP and non-AROP with the assessed maternal risk factors, including mode of delivery, comorbidities (hypertension and diabetes), cervical incompetence, and history of premature birth, premature rupture of membranes, and multiple pregnancy. This contrasts with some previous research that has suggested a potential link between maternal factors and ROP. For instance, studies by Mitra et al. [13] and Ahn et al. [14] reported an association between chorioamnionitis and ROP, albeit with conflicting results regarding its significance in multivariate analysis.

The absence of significant associations in our study could be attributed to various factors, including our sample size, which may have limited statistical power to detect subtle effects. Additionally, our study population primarily consisted of women from rural areas, potentially influencing the distribution of risk factors. Further research with larger, more diverse cohorts is needed to clarify the role of maternal factors in the development of AROP.

Our study revealed significant associations between AROP and prolonged NICU stay (>1 week), oxygen supplementation exceeding one week, and a longer duration of oxygen supplementation. These findings align with well-established knowledge like that of Kumar et al. and Kim et al. [11,12] that prematurity, low birth weight, and oxygen exposure are major risk factors for ROP. The duration of oxygen supplementation, in particular, has been consistently identified as a crucial factor in the development of severe ROP [15,16]. Our study reinforces the importance of minimizing oxygen exposure and optimizing neonatal care practices to reduce the risk of AROP.

In the present study, out of 107 eyes screened, treatment was given in 70 eyes (65.4%), and among them, regression and progression following treatment were noted in 52 (74.2%) and 18 (25.7%) eyes, respectively. However, treatment was not given for 14 (13%) eyes, and among them, regression and progression were noted in 10 (71.4%) and four (28.5%) eyes, respectively. Twenty-three eyes out of 107 (21.4%) were lost to follow-up due to family and economic issues.

We found a significantly higher rate of progression in cases with zone I AROP (28.5%) and spontaneous regression in cases with zone II posterior AROP (71.4%) without treatment (p<0.05). Though we found no significant association of outcome following treatment while analyzing individual zone-wise AROP (p>0.05), we found regression after treatment in significantly higher proportions of cases with zone I as compared to zone II posterior AROP (p<0.05). Among the treated eyes, out of 42 eyes that underwent laser, regression of AROP was noted in 76.1% (32 eyes), whereas 23.8% (10 eyes) progressed, of which 60% belonged to zone I and 40% to zone II posterior. Out of 16 eyes that underwent intravitreal anti-VEGF treatment, regression was noted in 75% (12 eyes), and 25% (four eyes) progressed. Eight out of 12 eyes (66.6%) that underwent combined treatment (laser+intravitreal anti-VEGF) regressed, but 33.3% (four eyes), 50% each from zone I and zone II posterior, progressed. Out of 70 eyes, 25.7% progressed to stage 4a/b and, hence, were referred to a higher centre for further management, consistent with previous studies by Sanghi et al. [17] where 71.4% regressed, 2.6% progressed to stage 5, 2.6% showed macular fold, and 23.4% showed peripheral tractional retinal detachment. Drenser et al. [18] have demonstrated the effectiveness of laser photocoagulation in treating APROP with 81.8% regression and only 18.2% progression to stage 4a/b. Similarly, Gunay et al. [19] found 93.3% of regression in eyes, whereas 6.7% progressed to stage 4a. Our analysis of treatment outcomes revealed variations in the effectiveness of different modalities based on the zone of AROP, the presence of plus or pre-plus disease, and the specific treatment combination. However, this finding may be attributed to the more aggressive nature of these cases, which may necessitate early and intensive treatment approaches. These findings highlight the importance of considering the severity of vascular abnormalities when selecting treatment modalities for AROP. Overall, our results underscore the need for personalized treatment approaches for AROP, taking into account the location of the disease, the presence of plus or pre-plus disease, and the individual patient's risk factors. Further research is needed to optimize treatment strategies and improve long-term visual outcomes for infants with AROP.

Limitations

The present study provides valuable insights into the course and outcome of AROP in our specific population. However, it is essential to acknowledge certain limitations that may influence the generalizability of our findings. Firstly, the prospective design of the study may have introduced potential biases, such as observer bias or dropouts. Secondly, the sample size, although substantial within our institution, may not be representative of all neonatal populations, particularly in diverse geographic regions or healthcare settings. Additionally, the study's focus on a single institution may limit the external validity of our findings, as treatment protocols, neonatal care practices, and patient demographics can vary across different centres. For instance, the high proportion of rural patients in our study may not reflect the distribution of AROP risk factors in urban populations, as highlighted by the differences in hospital birth rates and access to specialized care observed in studies.

## Conclusions

This study provides valuable insights into the course and outcome of AROP within our institutional context. The relatively high incidence of AROP, predominantly bilateral and affecting zone I, underscores the need for vigilant screening and early intervention in high-risk infants. Our findings reinforce the importance of minimizing exposure to supplemental oxygen and optimizing neonatal care practices to reduce the risk of AROP development and progression. The lack of significant associations with maternal risk factors highlights the multifactorial nature of AROP and the need for further research to unravel its complex etiology. Overall, this study contributes valuable insights to the existing body of knowledge on AROP, with implications for refining screening protocols, optimizing treatment algorithms, and ultimately improving visual outcomes for vulnerable preterm infants.
